# Phenotypic characterisation of regulatory T cells in patients with gestational diabetes mellitus

**DOI:** 10.1038/s41598-023-47638-z

**Published:** 2024-02-28

**Authors:** Ya-nan Zhang, Qin Wu, Yi-hui Deng

**Affiliations:** 1 School of Integrated Chinese and Western Medicine, Hunan University of Chinese Medicine, Hunan, 410208 China; 2 School of Chinese Medicine, Hunan University of Chinese Medicine, Hunan, 410208 China

**Keywords:** Endocrinology, Endocrine system and metabolic diseases

## Abstract

Gestational diabetes mellitus (GDM) is a common complication that occurs during pregnancy. Emerging evidence suggests that immune abnormalities play a pivotal role in the development of GDM. Specifically, regulatory T cells (Tregs) are considered a critical factor in controlling maternal–fetal immune tolerance. However, the specific characteristics and alterations of Tregs during the pathogenesis of GDM remain poorly elucidated. Therefore, this study aimed to investigate the changes in Tregs among pregnant women diagnosed with GDM compared to healthy pregnant women. A prospective study was conducted, enrolling 23 healthy pregnant women in the third trimester and 21 third-trimester women diagnosed with GDM. Participants were followed up until the postpartum period. The proportions of various Treg, including Tregs, mTregs, and nTregs, were detected in the peripheral blood of pregnant women from both groups. Additionally, the expression levels of PD-1, HLA-G, and HLA-DR on these Tregs were examined. The results revealed no significant differences in the proportions of Tregs, mTregs, and nTregs between the two groups during the third trimester and postpartum period. However, GDM patients exhibited significantly reduced levels of PD-1^+^ Tregs (*P* < 0.01) and HLA-G^+^ Tregs (*P* < 0.05) in the third trimester compared to healthy pregnant women in the third trimester. Furthermore, GDM patients demonstrated significantly lower levels of PD-1^+^ mTregs (*P* < 0.01) and HLA-G^+^ (*P* < 0.05) mTregs compared to healthy pregnant women in the third trimester. Overall, the proportion of Tregs did not exhibit significant changes during the third trimester in GDM patients compared to healthy pregnant women. Nevertheless, the observed dysregulation of immune regulation function in Tregs and mTregs may be associated with the development of GDM in pregnant women.

## Introduction

GDM is characterized by the occurrence or recognition of abnormal glucose tolerance during pregnancy^1^. The prevalence of GDM is rising globally and has been associated with epidemiological factors such as the rising rates of obesity among women of reproductive age and the advancing maternal age in recent decades^2,3^. GDM has detrimental effects on pregnancy outcomes and significantly increases the risk of metabolic disorders in both the offspring and the maternity^4^. GDM, being a multifactorial disease, is affected by a complex interplay of genetic, epigenetic, and environmental factors^5^. However, the precise pathogenic factors underlying GDM remains uncertain.

Regulatory T cells (Tregs) expressing the X chromosome-linked transcription factor Foxp3 represent a subset of CD4^+^ T cells that play a crucial role in controlling immune tolerance to self-antigens^6–8^. Upon stimulation by specific antigens, Tregs differentiate into CD45RO^+^ memory Tregs (mTregs) characterized by their long-term survival and rapid and effective immunoregulatory capacity. Specifically, CD45RO^+^ mTregs exhibit superior immunomodulatory and local immune migration capacities compared to CD45RA^+^ naive Tregs (nTregs) and exert a predominant immunosuppressive effect within the overall Tregs population^9,10^.

Emerging research suggests a potential association between GDM and maternal immune dysregulation^11,12^. The activation of immune cells in pregnant women, whether in the circulating peripheral blood or at the maternal infant interface, is considered an immune response directed towards the semi-allogeneic fetus^13^. Simultaneously, establishing maternal immune tolerance towards the fetus takes on critical significance in a successful pregnancy. When abnormalities arise in the number and immunoregulatory ability of mTregs, the delicate balance of immune responses in pregnant women will be disrupted, potentially leading to excessive maternal rejection of the fetus and the onset of GDM^11,12,14,15^.

However, the precise alterations involving mTregs in the context of GDM remain elusive, particularly regarding the expression of surface markers. This study aimed to investigate the composition of mTreg subgroups within the peripheral blood of both healthy pregnant women and GDM patients. Furthermore, we examined the presence of PD-1, HLA-G, and HLA-DR expression on the surfaces of Treg subsets. The overarching objective of this study was to uncover potential pathological immune mechanisms underlying GDM.

## Materials and methods

### Study population

In the present prospective study, a total of 23 healthy pregnant women and 21 women diagnosed with GDM were included, and their clinical data in the third trimester and postpartum period were investigated. The participants were recruited from the obstetrics department of the First Affiliated Hospital of Hunan University of Chinese Medicine between October 2022 and February 2023. All participants were within the age range of 25 and 35 and had no previous history of pregnancy, significant medical or surgical diseases, infections or metabolic disorders, autoimmune diseases, or chromosomal abnormalities. The healthy and GDM pregnant women had singleton pregnancies with no history of pregnancy loss or infertility. The diagnosis of GDM in patients was based on the following criteria: fasting blood glucose ≥ 5.1 mmol/l (92 mg/dl), 1-h blood glucose ≥ 10.0 mmol/l (180 mg/dl), or 2-h blood glucose ≥ 8.5 mmol/l (153 mg/dl) at 24 and 28 pregnancy weeks^16^. Other obstetric complications were not present in the GDM patients.

### Flow cytometry analysis

Blood samples were taken at room temperature in heparin anticoagulant tubes, then processed and analysed within 2 h. Tregs, mTregs and nTregs were analyzed with the following monoclonal antibodies (mAbs) from BioLegend (San Diego, California, USA): a blank control, a single standard control, PE anti-human CD25, APC anti-human HLA-G, PE/Cyanine7 anti-human CD279 (PD-1), Brilliant Violet 421™ anti-human HLA-DR, APC/Fire™ 750 anti-human CD45RO.

The operation was carried out in strict accordance with the manufacturer’s instructions (BioLegend, San Diego, California, USA), cells were treated with the corresponding mAbs for 15 min at room temperature in the dark for surface staining. Before being analyzed by a FACS Canto II flow cytometer (BD Biosciences, USA), cells were twice washed and suspended in PBS. In the aggregate, 100,000 events were recorded. The Tregs population were gated by CD3^+^, CD4^+^, CD25^+^, and CD127^low/−^. mTregs and nTregs were analyzed by adding CD45RO^+^ and CD45RA^+^ respectively to Tregs analysis. The PD-1, HLA-G, or HLA-DR expressing on Tregs subsets were gated afterward. Fluorescence minus one control were used to define negative and positive groups. The obtained data were exported to FlowJo v10.8.1 software for analysis.

### Statistical analysis

Data analysis was performed by using GraphPad Prism version 8.0.0. Categorical data are expressed as numerical values and percentages, and the chi-square (χ^2^) test was employed to examine their distribution. Numerical data are reported as mean ± standard error (SEM). The normality of the data distribution was assessed to determine if the data followed a normal distribution, and the probability of a random variable based on the dataset being normally distributed was calculated^17^. Differences between two groups were analyzed by utilizing Student's t-test, while group comparisons were conducted through one-way analysis of variance. Statistical significance was set at *P* < 0.05.

### Ethics statement

This study was approved by the Ethics Committee of the First Affiliated Hospital of Hunan University of Chinese Medicine and was conducted in accordance with the Declaration of Helsinki. All participants were fully informed and signed an informed consent before participating in the study. The datasets analysed in the current study are not publicly available due to the protection of patients' privacy and interests. However, they can be obtained upon reasonable request by contacting the corresponding authors.

## Results

### Clinical characteristics

A total of 44 participants were included in this prospective study, comprising 23 healthy pregnant women and 21 women diagnosed with GDM. The study focused on analyzing their clinical data from the third trimester to the postpartum period. Table 1 provides an overview of the participants’ clinical characteristics (e.g., age, BMI, gestation week of the participants).

**Table 1:** Clinical characteristics of normal pregnant women and GDM patients.

	Age (years)	BMI (kg/m^2^)	Gestation weeks	Postpartum days	Fasting glucose (mmol/L)	1 h OGTT (mmol/L)	2 h OGTT (mmol/L)
Healthy 3rd trimester	29.41 ± 0.73	29.77 ± 0.55	37.14 ± 0.44	–	4.42 ± 0.1	8.74 ± 0.12	7.76 ± 0.07
GDM 3rd trimester	29.06 ± 0.81	31.62 ± 0.42	37.38 ± 0.39	–	4.97 ± 0.05	9.69 ± 0.1	8.62 ± 0.09
Healthy postpartum	–	25.39 ± 0.38	–	46.11 ± 1.63	4.33 ± 0.09	–	–
GDM postpartum	–	27.82 ± 0.38	–	43.82 ± 1.27	4.91 ± 0.06	–	–

### The proportions of Tregs, mTregs and nTregs

**Figure 1:**
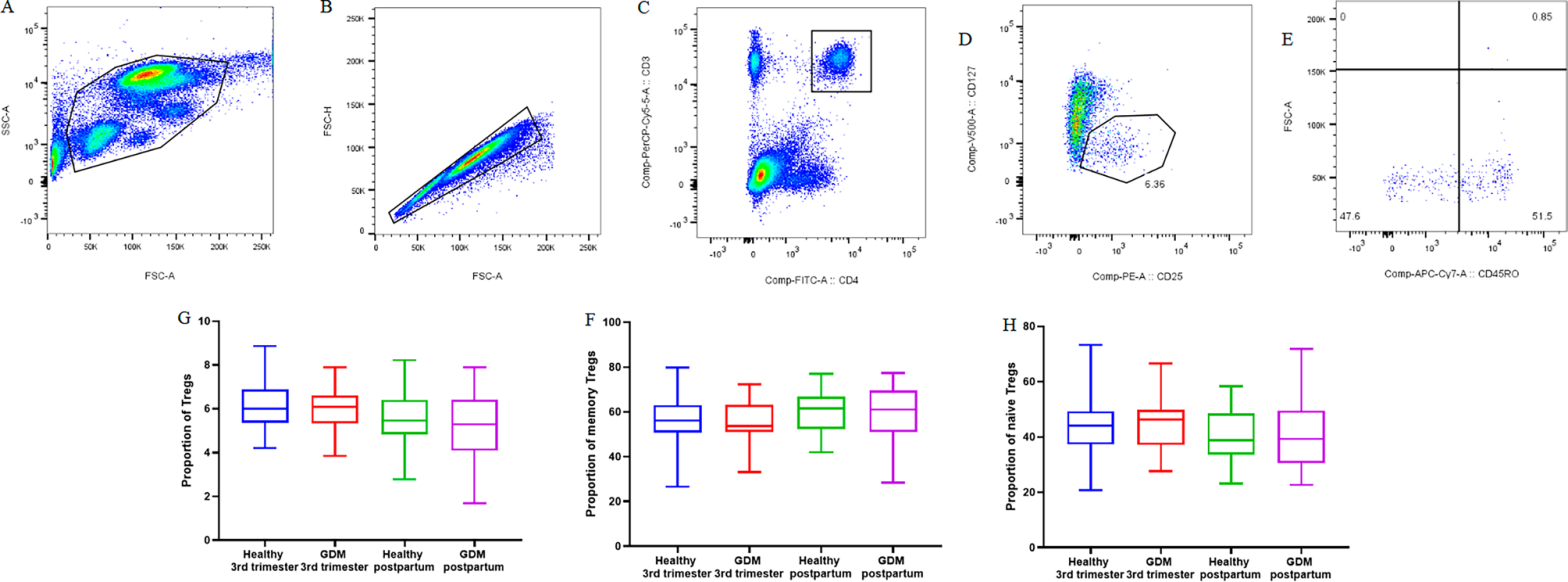
Gating strategy for Treg subsets determined by flow cytometry.

Figure 1 and Supplementary Table 1 illustrates the proportions of Tregs and mTregs in both pregnant women with GDM during the third trimester and healthy pregnant women during the same period. The results revealed no significant difference between the proportion of Tregs in the pregnant women with GDM during the third trimester (5.89% ± 0.22%) and healthy pregnant women in the third trimester (6.14% ± 0.26%, *P* = NS). Similarly, there was no significant difference in the proportion of mTregs between the pregnant women with GDM during the third trimester (55.70% ± 2.07%) and healthy pregnant women in the third trimester (56.70% ± 2.60%, *P* = NS).

Furthermore, the proportions of Tregs and mTregs were compared between GDM postpartum women and healthy postpartum women. The findings indicated no significant difference between the proportion of Tregs between the GDM postpartum women (5.13% ± 0.40%) and healthy postpartum women (5.67% ± 0.34%, *P* = NS). Likewise, there was no pronounced difference in the proportion of mTregs between the GDM postpartum women (58.50% ± 3.38%) and healthy postpartum women (60.80% ± 2.23%, *P* = NS).

Overall, no significant differences were observed in the proportions of Tregs, mTregs, nTregs between healthy women and GDM patients during the postpartum compared to the third trimester of pregnancy.

### Expression level of PD-1, HLA-G, HLA-DR on Tregs in healthy pregnancy and GDM

**Figure 2:**
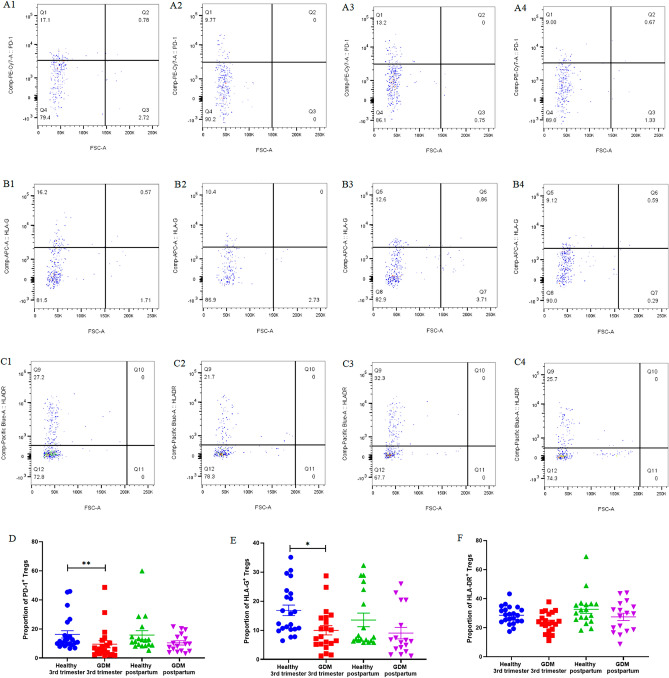
The expression of PD-1, HLA-G, and HLA-DR on Tregs. Healthy third-trimester women: (**A1**), (**B1**), (**C1**). GDM third-trimester women: (**A2**), (**B2**), (**C2**). Healthy postpartum women: (**A3**), (**B3**), (**C3**). GDM postpartum women: (**A4**), (**B4**), (C4).

As depicted in Fig. 2 and Supplementary Table 2, the expression of PD-1 on Tregs in pregnant women with GDM during the third trimester (9.58% ± 2.47%) was significantly lower than that in healthy pregnant women in the third trimester (16.31% ± 2.53%, *P* < 0.01). The expression of HLA-G on Tregs in pregnant women with GDM during the third trimester (10.01% ± 1.57%) was lower than that in healthy pregnant women in the third trimester (16.86% ± 1.80%, *P* < 0.05). There was no significant difference in the expression level of HLA-DR on Tregs between the pregnant women with GDM during the third trimester (23.69% ± 1.49%) and healthy pregnant women in the third trimester (28.55% ± 1.30%, *P* = NS).

There was no significant difference in the expression level of PD-1, HLA-G, HLA-DR on Tregs between GDM patients and healthy women in postpartum. Moreover, there was no significant difference in the expression level of PD-1, HLA-G, HLA-DR on Tregs between the postpartum and third-trimester, whether in healthy women and GDM patients.

### Expression level of PD-1, HLA-G, HLA-DR on mTregs in healthy pregnancy and GDM

**Figure 3:**
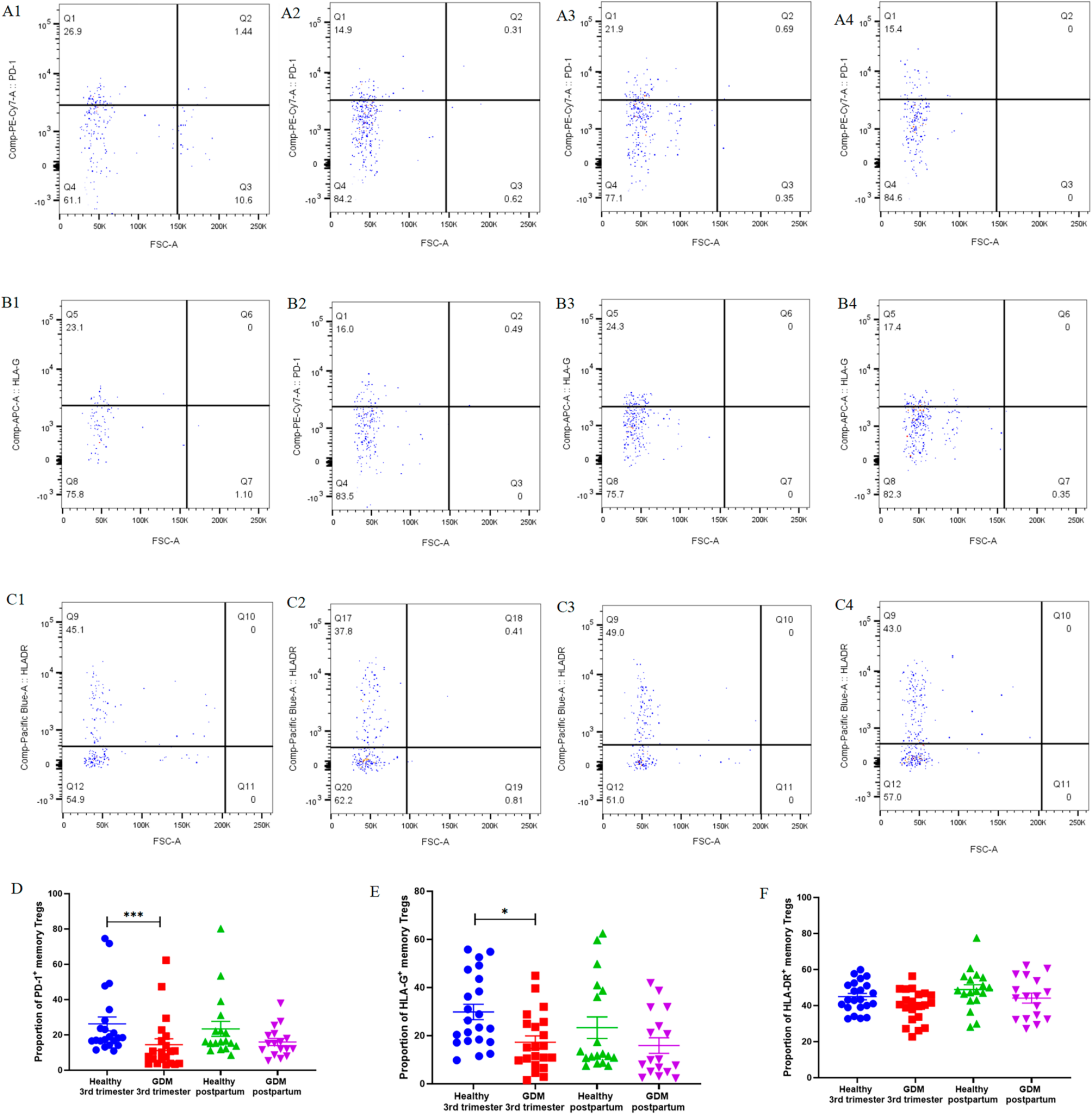
The expression of PD-1, HLA-G, and HLA-DR on mTregs. Healthy third-trimester women: (**A1**), (**B1**), (**C1**). GDM third-trimester women: (**A2**), (**B2**), (**C2**). Healthy postpartum women: (**A3**), (**B3**), (**C3**). GDM postpartum women: (**A4**), (**B4**), (**C4**).

As depicted in Fig. 3 and Supplementary Table 2, the expression of PD-1 on Tregs in pregnant women with GDM during the third trimester (14.45% ± 3.34%) was significantly lower than that in healthy pregnant women in the third trimester (26.24% ± 3.91%, *P* < 0.01). The expression of HLA-G on Tregs in pregnant women with GDM during the third trimester (17.27% ± 2.60%) was lower than that in healthy pregnant women in the third trimester (29.85% ± 3.19%, *P* < 0.05). There was no significant difference in the expression level of HLA-DR on Tregs between the pregnant women with GDM during the third trimester (39.83% ± 1.94%) and healthy pregnant women in the third trimester (44.98% ± 1.78%, *P* = NS).

There was no significant difference in the expression level of PD-1, HLA-G, HLA-DR on mTregs between GDM patients and healthy women in postpartum. Moreover, there was no significant difference in the expression level of PD-1, HLA-G, HLA-DR on mTregs between the postpartum and third-trimester, whether in healthy women and GDM patients.

### Expression level of PD-1, HLA-G, HLA-DR on nTregs in healthy pregnancy and GDM

**Figure 4:**
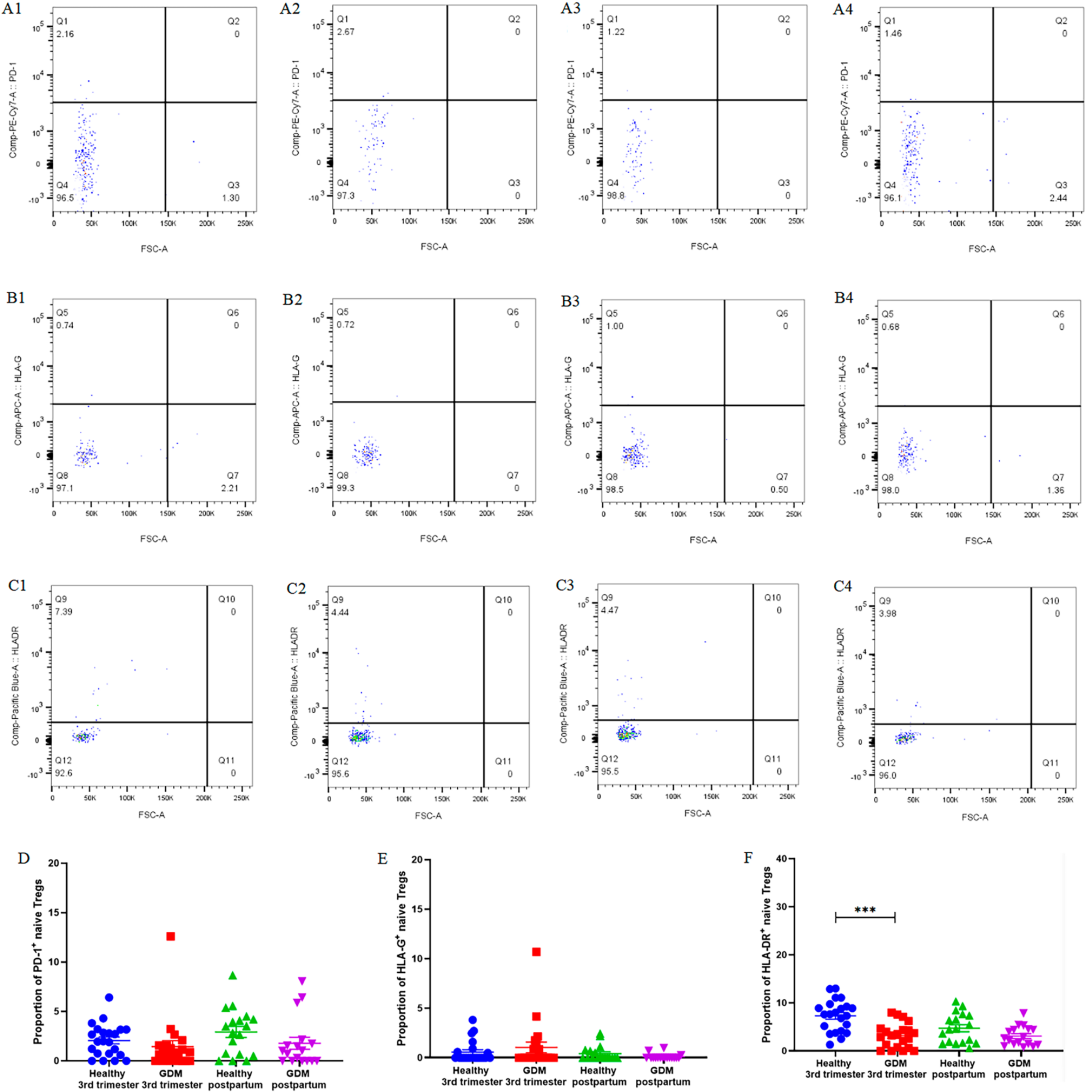
The expression of PD-1, HLA-G, and HLA-DR on nTregs. Healthy third-trimester women: (**A1**), (**B1**), (**C1**). GDM third-trimester women: (**A2**), (**B**2), (**C2**). Healthy postpartum women: (**A3**), (**B3**), (**C3**). GDM postpartum women: (**A4**), (**B**4), (**C4**).

As depicted in Fig. 4 and Supplementary Table 2, there was no significant difference in the expression level of PD-1, HLA-G, HLA-DR on nTregs between the postpartum and third-trimester, whether in healthy women and GDM patients.

There was no significant difference in the expression level of PD-1 and HLA-G on nTregs between the healthy third-trimester women and GDM third-trimester women. The expression of HLA- DR on nTregs in GDM third-trimester women (3.19% ± 0.56%) was significantly lower than that in healthy third-trimester women (7.32% ± 0.70%, *P* < 0.01).

There was no significant difference in the expression level of PD-1, HLA-G, HLA-DR on nTregs between GDM postpartum women and healthy postpartum women.

## Discussion

Recent research indicated that maintaining a relative immune balance between immune activation and suppression is crucial for a healthy pregnancy^18,19^. A developing fetus is considered an allograft within in the immunocompetent maternal host^20^.

Prior to conception, the maternal immune system encounters antigens from the paternal sperm. During embryo implantation, paternal antigens present on placental trophoblasts directly interact with immune cells at the maternal–fetal interface. This interaction can trigger an inflammatory response that has the potential to disrupt the success of pregnancy.

The establishment of maternal immune tolerance is pivotal for a successful pregnancy, and this process is primarily mediated by CD4^+^ Tregs^21^. Tregs have an important effect in improving fetal growth and survival by suppressing the maternal immune system from recognizing the semi-allogeneic carrying paternal antigens^7,21^. CD25^high^ FOXP3^+^ Tregs become dominant in decidual tissues during pregnancy and contribute to the establishment of maternal immune tolerance to invading fetal extravillous trophoblasts^22^. Abnormalities in number and function of Tregs may be associated with various pregnancy complications, including GDM^23–27^.

Upon stimulation by specific antigens, CD45RA^+^ nTregs differentiate into CD45RO^+^ mTregs, which exhibit long-term survival and rapid and effective immunoregulatory capacity. The recruitment of a substantial number of mTregs to the maternal–fetal interface tissues are advantageous in suppressing acute allograft immune response to the fetus^9^.

GDM is a prevalent medical complication during pregnancy and is associated with various adverse maternal and fetal pregnancy complications^28–30^. Recent research has suggested that GDM is a disease marked by chronic systemic inflammation and an elevated humoral immune response^31,32^. Tregs take an important role in suppressing excessive inflammation of the immune system^33,34^. It has been reported that the high-proliferative but dysfunctional mTregs in the peripheral blood result in diabetes in NOD mice^35^. Furthermore, type 1 diabetic patients may be associated with defective inhibitory function of mTregs caused by low expression of CD39^36^. The development and function of both naive and memory Treg populations are modified in GDM patients, including a lower expression of suppressive Treg subsets such as CD4^+^CD127^low+/−^CD25^+^ Tregs and CD45RA^−^ Tregs^14^.

In this study, no significant differences were found in the percentage of Tregs between GDM patients and healthy pregnant women, whether in the third-trimester or postpartum. The above-mentioned finding aligns with previous research results, indicating that GDM patients may not experience significant changes in the proportion of Tregs, but abnormalities in the composition and function of Tregs^14^. Furthermore, there is no significant difference in the proportion of mTregs and nTregs in the peripheral blood of GDM patients in the third trimester compared to healthy pregnant women in the third trimester in this study. There is also no statistically significant change in the proportion of mTreg and nTreg in the peripheral blood of GDM postpartum women versus healthy postpartum women. This may be due to the fact that the establishment of immune memory of Tregs is affected by metabolic factors besides the conventional immune process. As a matter of fact, proliferation is dependent on glycolysis, and memory is dependent on fatty acid oxidation, as suggested by previous studies^37^. However, whether the increasing in memory Tregs is associated with metabolism should be explored further. Besides, Subsequent studies with larger sample sizes are needed because such results may also be limited by the small number of participants.

Immune checkpoints serve as a group of suppression pathways expressed as ligand receptors on the surface of a wide variety of immune cells. The expression of immune checkpoints (e.g., CTLA-4, PD-1, TIM-3, and LAG-3) allows immune cells not only to maintain self-tolerance, but also to regulate the intensity of the immune response^38,39^. The characteristic expression of immune checkpoint molecules on the surface of Tregs or mTregs may imply a modification of their proliferation ability and functionality^40^. The most recent data indicates that they have a strong relationship with pregnancy outcomes through a variety of inhibitory mechanisms^40,41^.

The PD-1/PD-L1 pathway is an important immune regulatory pathway that can entirely suppress the immune reaction through the reduction of T cell proliferation, the increase of T cell anergy and fatigue, the reduction of cytokine production and the activation of Tregs^42^. Tregs expressing a high level of PD-1 have the ability to suppress Teff function and proliferation in an IL-10 dependent way^43^. It is also possible to use the PI3K/Akt/mTOR pathway to accomplish the role of the PD-1/PD-L1 pathway in suppressing T cell activation^44,45^. The dynamic equilibrium of mTregs can be suppressed by PD-1, and anti-PD-1 therapy can play an important role in interfering with the accumulation of mTregs.

In this study, we observed a significant decrease in the expression of PD-1 on Tregs and mTregs in pregnant women with GDM during the third trimester compared to healthy third-trimester pregnancies. There was no significant difference in the proportion PD-1^+^nTregs between healthy third-trimester pregnancies and GDM in the third trimester. This indicates that Tregs and mTregs as essential factors of maternal–fetal immune tolerance, the low-level expression of PD-1^+^ Tregs and mTregs in the peripheral blood of GDM patients may result in immune imbalance and is associated with the pathogenesis of GDM. Previous studies have found that the proportion of PD-1^+^ Tregs in the peripheral blood of healthy pregnant women in the third trimester was higher than that of GDM patients, suggesting that the expression of PD-1 on T cell subsets can serve as a vital marker for the occurrence and recovery of GDM^46–48^. Nevertheless, there was no significant difference in the expression level of PD-1 on Tregs, mTregs, nTregs between GDM postpartum women and healthy postpartum women in this study. Whether in GDM patients or healthy pregnant women, the proportion of postpartum PD-1^+^ Tregs and PD-1^+^ mTregs was not significantly lower than in third-trimester, indicating that Tregs can still play an immune regulatory role for a period of time after delivery, rather than apoptosis quickly^49^. Further investigation using prospective studies with larger sample sizes is needed to gain a better understanding of the changes in Tregs between the third trimester and the postpartum period.

The non-classical major histocompatibility complex (MHC) class I molecule HLA-G is regarded with playing a significant role in this unique immune suppression system during pregnancy^50^. HLA-G expression is dependent on Enhancer L, a cis-regulatory element located 12 kb upstream of the HLA-G locus^51^. HLA-G could not only suppress CD4^+^ T cell proliferation, decrease CD4^+^ T cell immune responsiveness, but also facilitate T cell differentiation into Treg^52,53^. Additionally, Tregs expressing HLA-G could secrete substantial inhibitory chemicals (such as soluble HLA-G and IL-10) or use cell–cell non-contact machinery to inhibit T-cell reactions^54,55^.

CD4^+^ T cells exhibited an increased differentiation tendency of Tregs after being stimulated by HLA-G^+^ EVT^56^. These Tregs remained after childbirth and were seen in the following pregnancy. Studies have reported that women having higher sHLA-G levels in their peripheral blood had a greater possibility of having a successful IVF therapy^57,58^. Pregnancy complications (e.g., miscarriage, preterm delivery, preeclampsia, and recurrent pregnancy loss(RPL)) have been reported to be associated with naturally occurring HLA-G polymorphisms that may trigger lower HLA-G levels^51,59,60^. In a previous study, the level of HLA-G in placental extravillous trophoblasts was lower in patients with GDM than in normal controls^61^. In the present study, the proportion of HLA-G^+^ Tregs and mTregs in third-trimester women diagnosed with GDM were lower than that in healthy third-trimester pregnancies. The results above demonstrate that the reduced expression of HLA-G on Tregs and mTregs' surface could hinder the inhibition of harmful maternal alloresponsiveness. Moreover, an overactive immune system may disrupt the mother's natural physiological balance, potentially leading to GDM. However, no significant differences were reported in the expression level of HLA-G on Tregs, mTregs, nTregs between GDM postpartum women and healthy postpartum women. Larger samples and more in-depth analyses are required for elucidating the reasons for the above results.

HLA-DR has been considered an activation marker expressed by CD4^+^ T lymphocytes when stimulated by related antigens^62^. Furthermore, Foxp3-expressing HLA-DR^+^ Tregs have a quicker and more powerful immunosuppressive effect on T lymphocytes^63^. Existing research has indicated a noteworthy reduction in the proportion of naive CD45RA^+^ Tregs in individuals suffering from dietary-adjusted GDM as well as those grappling with insulin-dependent GDM^14^. HLA-DR^+^ mTregs were significantly decreased in patients with diet-dependent GDM, while significantly increased in patients with insulin-dependent GDM^14^. In this study, the proportion of HLA-DR^+^ Tregs and HLA-DR^+^ mTregs in pregnant women with GDM during the third trimester was slightly lower than that in healthy pregnant women in the third trimester. The proportion of HLA- DR^+^ nTregs in third-trimester women with was significantly lower than that in healthy pregnant women in the third trimester. Besides, the proportion of HLA-DR^+^ Tregs, HLA-DR^+^ mTregs, HLA-DR^+^ nTregs in GDM postpartum women was slightly lower than that in healthy postpartum women. This suggests that the inhibitory capacity of Tregs is partially impaired in patients with GDM compared to healthy pregnancies. As an activation marker of T lymphocytes, whether the expression of HLA-DR in Tregs subgroups is associated with the pathogenesis of GDM needs to be further investigated.

However, this study has several limitations. Firstly, the sample size was small, with only 44 participants, thus requiring subsequent enlargement to improve the results' reliability. Secondly, it solely focused on Tregs in peripheral blood without exploring their characteristics at the maternal–fetal interface. Finally, this study solely focused on the Tregs population. A more comprehensive analysis of other immune cells could have provided more significant conclusions.

## Conclusion

In conclusion, there is no significant difference in the proportion and composition of Tregs between healthy pregnant women and GDM patients. However, during the third-trimester, the expression of functionally related factors such as PD-1 and HLA-G on Tregs and mTregs in GDM patients is lower compared to healthy pregnant women in the third trimester. This indicates that the dysfunction of Tregs and mTregs may be associated with the pathogenesis of GDM. However, this study had a limited number of participants, and further in-depth exploration require prospective research with a larger sample size.

### Supplementary Information

Supplementary Tables.

## Data Availability

The data that support the findings of this study are available on request from the corresponding author Y.D. upon reasonable request.
