# Supplementary material for: Phenotypic characterisation of regulatory T cells in patients with gestational diabetes mellitus
Source: Sci Rep. 2024 Feb 28;14:4881. doi: 10.1038/s41598-023-47638-z (PMC10902321; doi:10.1038/s41598-023-47638-z)
Supplement: Supplementary file 1 — Supplementary Tables. [file 41598_2023_47638_MOESM1_ESM.docx]

Supplementary Table 1. Proportion of Tregs, mTregs and nTregs.

|  | Healthy  3rd trimester | GDM  3rd trimester | Healthy postpartum | GDM postpartum |
| --- | --- | --- | --- | --- |
| Tregs/CD4^+^ T cells | 6.14±0.26 | 5.89±0.22 | 5.67±0.34 | 5.13±0.40 |
| mTregs/Tregs | 56.70±2.60 | 55.70±2.07 | 60.80±2.23 | 58.50±3.38 |
| nTregs/Tregs | 43.50±2.58 | 44.50±2.06 | 39.60±2.24 | 41.90±3.40 |

Supplementary Table 2. Expression level of PD-1, HLA-G, HLA-DR on Tregs, mTregs and nTregs.

|  | Healthy  3rd trimester | GDM  3rd trimester | Healthy postpartum | GDM postpartum |
| --- | --- | --- | --- | --- |
| PD-1^+^ Tregs | 16.31±2.53 | 9.58±2.47^※※^ | 15.86±3.03 | 10.50±1.58 |
| HLA-G^+^ Tregs | 16.86±1.80 | 10.01±1.57^※^ | 13.57±2.34 | 9.06±2.10 |
| HLA-DR^+^ Tregs | 28.55±1.30 | 23.69±1.49 | 32.64±2.76 | 27.43±2.69 |
| PD-1^+^mTregs | 26.24±3.91 | 14.45±3.34^※※^ | 23.42±4.25 | 15.98±2.17 |
| HLA-G^+^ mTregs | 29.85±3.19 | 17.27±2.60^※^ | 23.31±4.50 | 15.89±3.46 |
| HLA-DR^+^ mTregs | 44.98±1.78 | 39.83±1.94 | 48.90±2.68 | 44.12±2.96 |
| PD-1^+^ nTregs | 2.06±0.35 | 2.93±0.56 | 1.46±0.59 | 1.79±0.65 |
| HLA-G^+^ nTregs | 0.57±0.23 | 0.43±0.18 | 1.05±0.53 | 0.12±0.07 |
| HLA-DR^+^ nTregs | 7.32±0.70 | 3.19±0.56^※※^ | 4.74±0.73 | 3.09±0.52 |

^※^ ^※^Compared with healthy 3^rd^ trimester, P＜0.05

^※※^ Compared with healthy 3^rd^ trimester, P＜0.01
